# “The Terrors of Kingly Power”: The Unusual Dental Pathology of King Pyrrhus of Epirus

**DOI:** 10.7759/cureus.42356

**Published:** 2023-07-24

**Authors:** Matthew D Turner, Michael J Lawson, Kevin D Smith

**Affiliations:** 1 Emergency Department, Penn State Health Milton S. Hershey Medical Center, Hershey, USA; 2 Dermatology Department, Brooke Army Medical Center, San Antonio, USA; 3 Oral and Maxillofacial Surgery Department, Madigan Army Medical Center, Lakewood, USA

**Keywords:** history, pyrrhus, calculus overgrowth, developmental dysplasia, dental anomalies, ancient medicine

## Abstract

Pyrrhus of Epirus, widely respected and feared by his contemporaries, was a legendary figure in the ancient world. In this paper, we investigate Plutarch’s description of the king’s unique dental pathology. There are several possibilities to explain the ancient king's presentation, including several different types of developmental dysplasia. However, our conclusion is that it was likely due to a significant dental calculus overgrowth, often seen in the ancient Greek diet of the time. Whatever the underlying cause, Pyrrhus' intimidating visage helped secure the king a legacy that lasts to this day.

## Introduction and background

King Pyrrhus of Epirus, who lived from 319 to 272 BCE [1], is a fascinating historical figure. From a young age, the Greek king was a man of “burning ambition, and almost totally absorbed by fighting and military matters” [2]. He found peace “tedious to the point of nausea” [2], and was desperate to secure a legacy as a conqueror comparable to that of Alexander the Great, to whom he was a distant cousin [3]. Throughout his tumultuous reign, he secured a large and powerful kingdom that soon came into conflict with the growing power of Rome on the Italian peninsula. With a massive invasion force consisting of thousands of cavalry, tens of thousands of infantry, and 20 elephants, he defeated the Romans in several major engagements throughout the 270s BCE [1]. However, his losses in these victories were so great that they ultimately doomed his Italian campaign, providing the origin of the expression “pyrrhic victory” [2]. This was further worsened by Pyrrhus’s consistent inability to consolidate his victories [2]. The Romans quickly recovered from their losses and conquered the last of the Greek colonies in Italy. Just a few years after his death, Pyrrhus died an ignoble death, “struck on the head by a tile thrown from the roof of a house” [1]. Despite his military talents and pursuit of military excellence “to the exclusion of all else,” he was never able to achieve his goal of becoming a new Alexander [3].

Most of the information regarding Pyrrhus available in the modern age was recorded by Roman historians-writers who often wrote more “to moralize and to sustain the readers’ interest (than) give an accurate chronological account of events” [4]. Although Roman sources are generally more sympathetic to Pyrrhus than other historical opponents of Rome, such as Hannibal, the Greek historian Plutarch’s account is widely regarded as less biased and more historically accurate than other sources available [4]. In Plutarch’s Lives, the historian describes the king’s imposing features: “Pyrrhus in the air of his face had something more of the terrors than of the augustness of kingly power; he had not a regular set of upper teeth, but in the place of them one continued bone, with small lines marked on it, resembling the divisions of a row of teeth” [5]. This unusual appearance further added to Pyrrhus’ reputation as a powerful and intimidating warrior [2]. In this paper, we seek to explain the possible causes of the ancient king’s unusual dental pathology.

## Review

Possible explanations

Mythologizing

Pyrrhus himself appears to have had something of an oral fixation. In one example, on the eve of an important battle, he supposedly “was subjected to nightmares where most of his teeth fell out and he bled from the mouth” [2]. He considered the dream to be an ill omen and attempted to delay the engagement, but was persuaded not to by his advisors. The subsequent battle ended up being a disastrous defeat [2]. Although this story may be apocryphal, it does emphasize how Pyrrhus’ emphasis on certain parts of his body as having special qualities contributed to his mythologizing. In addition to Plutarch’s description of the king’s unusual teeth [5], Pyrrhus himself claimed to have a “divine virtue” of the “great toe of his right foot” that he could use to miraculously cure the spleens of those he touched with the digit [2]. Later Western monarchs would similarly claim a special “King’s Touch” that they could use to cure maladies such as scrofula [6]. By ascribing special qualities to his body, Pyrrhus would have further secured his grasp on the reins of power-something likely always foremost on his mind, for he had briefly lost the throne as a child [1]. In this context, Pyrrhus’ unusual dental pathology may be simply propaganda meant to impress and intimidate. Plutarch's descriptions of the "terrors... of kingly power" visible on the would-be conqueror's visage [5] would have thus played exactly into the image that Pyrrhus wished to present to the world.

It is also important to note that Plutarch’s description of Pyrrhus was written several centuries after the king’s death [2]. Other ancient sources, such as Ianuarius Nepotianus’s works, that discuss the unique signum regalitatis of Pyrrhus’ unusual dental pathology are similarly dated [7]. Even during his own lifetime, Pyrrhus employed lackeys such as Proxenus, an “artfully chosen historian,” to spread mythologizing tales of himself “for purposes of advertisement and diplomacy” [8]. Unfortunately, most of the contemporary literature regarding Pyrrhus has been lost; later histories had to draw extensively on the “traces” left behind by contemporary authors such as Timaeus [9]. Even Pyrrhus’ own memoirs have been lost to time [10].

However, while Pyrrhus may have exaggerated his dental pathology for the purposes of propaganda, it is unlikely that either he or his subsequent biographers completely fabricated the story. As discussed earlier, Plutarch is widely regarded as a reliable source regarding the king [4]. Furthermore, it would have been impractical for Pyrrhus to spread rumors of such an unusual dental pathology when he routinely met his enemies face-to-face at diplomatic events [2]. This leads us to suspect that there was a biological aspect-not just propaganda and mythologizing-at play in Pyrrhus’ dental presentation.

A Possible Developmental Dysplasia

It has been suggested that Pyrrhus’ dental pathology was due to an unspecified developmental cause [2]. While there are many inherited conditions that may interfere with tooth development, such as hypophosphatasia, taurodontism, amelogenesis imperfecta, and dozens of other gene networks that “regulate the development of teeth,” the majority present with either hypodontia, familial tooth agenesis, or “premature exfoliation of primary teeth” [11]. None of these are consistent with the description of Pyrrhus’ dental pathology [2].

**Figure 1:**
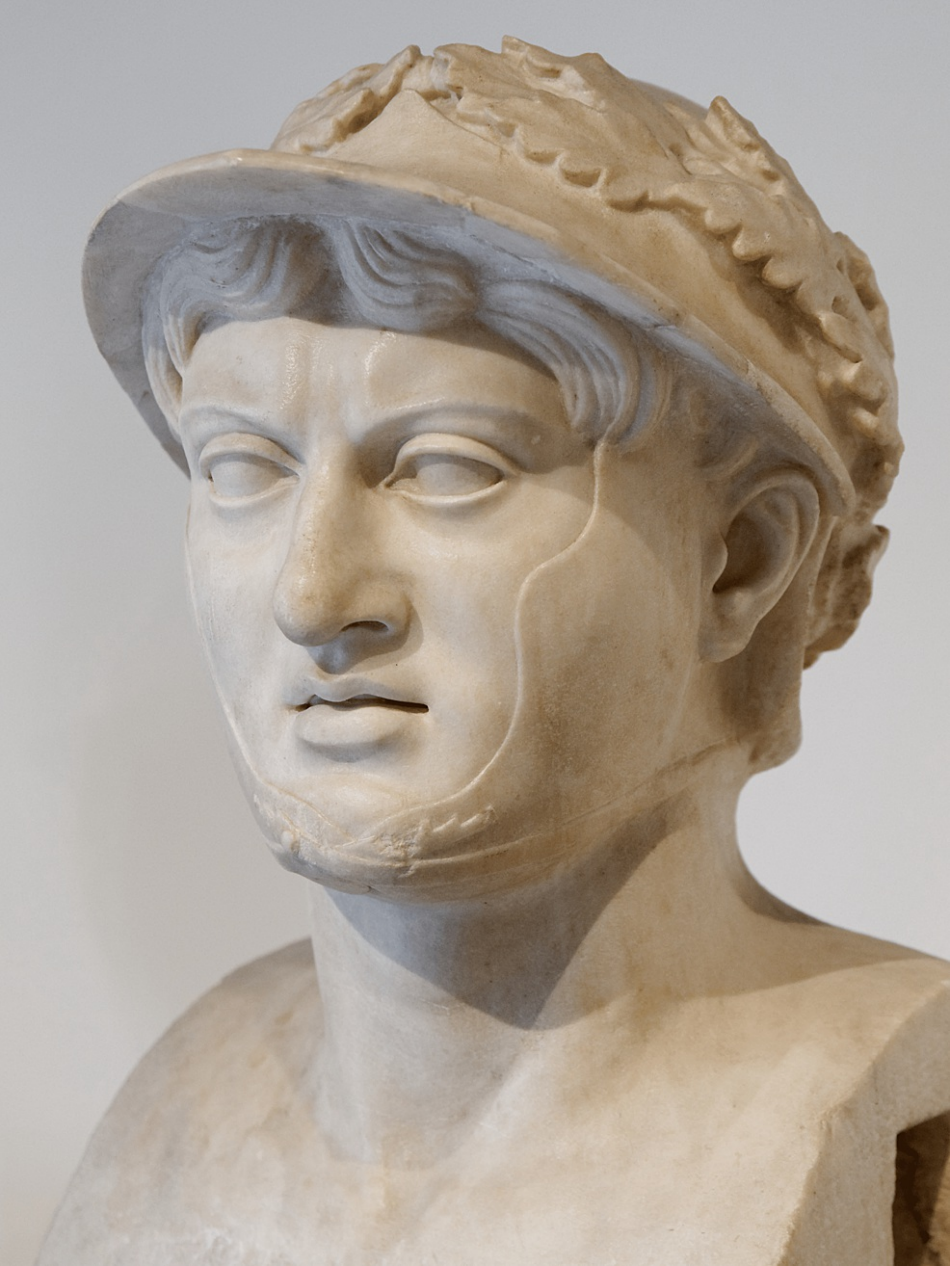
A Roman bust of Pyrrhus, circa 50–25 BCE, copied from an earlier Greek bust. This picture is part of the public domain. Source: [14].

Figure 1 depicts a Roman bust of Pyrrhus from approximately 50-25 BCE. While not contemporary with the monarch, it was copied from a Greek original [9]. The bust does not display Pyrrhus’ teeth directly, but it does display mild frontal bossing, a potential clue to an underlying diagnosis. Frontal bossing may be associated with a number of systemic conditions, including acromegaly, fragile X syndrome, and extramedullary hematopoiesis [12]. However, in a number of ectodermal syndromes, frontal bossing may also be associated with dental abnormalities [13].

Given the mild frontal bossing on the king, we suggest that the Pyrrhus’ unusual presentation may have been due to an underlying ectodermal syndrome, such as anhidrotic ectodermal dysplasia, a rare developmental abnormality that is characterized by “abnormal development of sweat glands, teeth, and hair” [13]. The teeth in affected patients may present with oligodontia as well as frontal bossing [15], similar to that seen in Figure 1. Similarly, chondroectodermal dysplasia may have significant oral manifestations in line with the descriptions of Pyrrhus’ teeth, including hypertrophy of the frenulum, hypodontia, and cleft palate [16].

**Figure 2:**
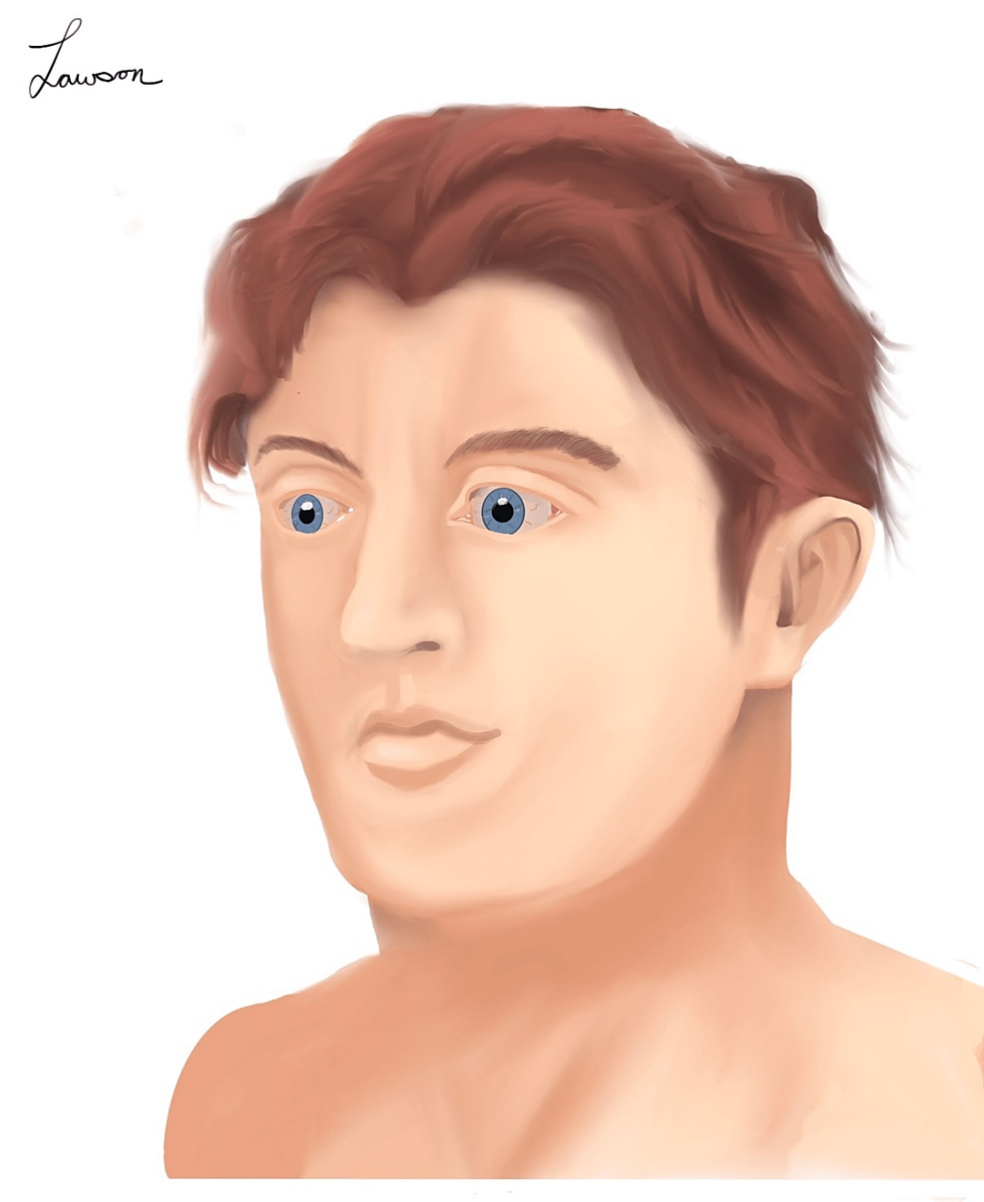
A contemporary illustration of Pyrrhus based on historical descriptions of the king. This medical illustration was created by Dr. Michael Lawson, the author of this paper.

However, while these are interesting possibilities, it is unlikely that either of these ectodermal pathologies are responsible for Pyrrhus’s dental condition. While it is impossible to determine if Pyrrhus experienced any form of anhidrosis or hypohidrosis consistent with anhidrotic ectodermal dysplasia [13], he likely did not experience the hypotrichosis that would be expected from this pathology [13]. The bust shown in Figure 1 clearly shows that Pyrrhus had a full head of hair beneath his helmet. In addition to this, the etymology of the name Pyrrhus typically meant “flame-colored” or “red-headed.” Although this may have been intended to link the king with the destructive power of fire [17], it is possible that it could have referred to his hair color as well. Whatever the case, it appears highly unlikely that the monarch suffered from anhidrotic ectodermal dysplasia. Based on this description and the bust in Figure 1, we propose that the king resembled the illustration seen in Figure 2.

Chondroectodermal dysplasia is also unlikely. This dysplasia is strongly associated with congenital cardiac abnormalities, ectodermal dysplasia, and polydactyly [16], none of which can be found in the historical record surrounding the king. If Pyrrhus, a man famous to his contemporaries for claiming to have divine healing powers in his right great toe [2] had been born with an extra digit, it is a near certainty that it would have been recorded, if for no other reason than to add to his near-insatiable desire to secure a legacy [2].

While the frontal bossing displayed by Pyrrhus’ bust initially suggests an underlying syndrome that may explain the description of his dental abnormality, our theory falls apart on closer inspection. None of the descriptions of Pyrrhus support any sort of systemic dysplasia. In addition to this, the mild frontal bossing displayed on his bust may be consistent with the typical male facial structure, which is associated with a more prominent supraorbital ridge and a more prominent glabella [18]. Given this, Pyrrhus’ frontal bossing may have been simply a benign phenotypic variant, or perhaps even an artistic choice to present the king with a heavy brow-line, consistent with contemporary imagery of mythological figures such as Herakles [19,20].

Vertical Maxillary Excess

With developmental dysplasia unlikely to be responsible, vertical maxillary excess (VME) emerges as an alternative explanation. Often known as a “gummy smile” in the modern age, this common orthognathic issue is due to a disproportionately vertical maxilla [21]. Other possible causes of a “gummy smile” include an abnormal eruption of the teeth from the gumline manifesting as a shortened clinic crown and hyperactivity of the upper lip muscles [22]. In our clinical experience, significant VME can cause the teeth to be almost completely obscured by the lower lip. In extreme cases, only the dental alveolus and nasomaxillary/pterygomaxillary buttresses are observable, and could be described as appearing as “one continuous bone” like in Plutarch’s writings [5].

However, Pyrrhus’ bust in Figure 1 again rules against this. VME is strongly associated by excessive lower vertical facial height [23], a phenomenon that researchers have dubbed “long face syndrome” [24]. Patients with “long face syndrome” often display a narrow nose, depressed nasolabial areas, and a long lower third of the face [24], none of which can be observed in Figure 1. Given that Pyrrhus’ remains were cremated [2], it is impossible to conclusively determine if he had VME. However, the best evidence currently available to us strongly suggests against it.

Heavy Calculus Deposition

Jeff Champion, one of the prominent modern biographers of Pyrrhus, has proposed that Pyrrhus’ dental pathology may have been simply due to heavy calculus deposition [2]. Pyrrhus may have simply suffered from heavy calculus deposition. In this process, typically associated with a lack of professional dentistry or regular oral hygiene, the dental plaque can calcify [25]. Formed on the teeth “through a complex interaction between saliva and bacteria on the dental surface” [26], dental calculus has significant “preservation potential”, and can be found in remains as old as 8-12 million years old [26]. Of note, dental plaque is formed secondary to the metabolization of salivary sugars by a number of anaerobic bacteria, *Streptococcus mutans* being chief among them. This metabolic process forms a film that adheres to the dental surface; if not cleaned within a period as short as two weeks, the plaque rapidly hardens [26]. This process occurs in a positive feedback loop, as “the rough surface of dental calculus serves to attract other bacteria that adhere to those already attached” [26].

In a study of Apollonia, a Greek colony located on the Black Sea, researchers found remains from the fifth to second centuries BCE that displayed evidence of dental calculus in 79.1% of the 158 individuals examined. Notably, older adults were significantly more likely to display dental calculus [27]. While multiple other factors affect the rate of dental caries, including genetic factors, mineral content, and salivary flow, the current data suggest that a high protein intake is associated with a lower incidence of caries, with the opposite holding true for a carbohydrate-rich diet [26]. The typical carbohydrate-heavy Greek diet of the time had a heavy emphasis on soft foods made from cereal crops. This, in conjunction with poor oral hygiene, likely exacerbated calculus buildup [27]. This “Mediterranean triad” of cereals, olive oil, and wine, practiced by both the ancient Roman and Greek civilizations often precluded caries and other dental pathologies [28]. Pyrrhus died at approximately the age of 46 [2]; given this, it is possible that he may have had dental calculus present, as found in the Apollonia study [27]. Dentistry was not unknown to the ancient Mediterranean population-there is evidence that the Egyptians employed professional dentists over 2000 years before Pyrrhus’ birth [29]. However, it is unlikely that Pyrrhus would have had access to routine professional care, given his love of the battlefield and the years he spent away from home on various military campaigns [2].

While it is usually restricted to the “lingual surfaces of incisors and canines and the buccal surfaces of maxillary molars,” dental calculus can “almost cover all teeth” in the most extreme cases [26]. There is precedent in the literature for such significant dental calculus growth that it may present in “giant” form [30,31]. One 2008 case report of an 82-year-old British woman described a severe dental calculus so significant that it resembled a bony tumor mass [32]. Although the woman in question only had two remaining teeth at the time, the calculus deposits entirely covered the dental surface, so much so that the teeth could only be found within the hard tissue mass through x-ray [32]. Such examples demonstrate that significant calculus overgrowth is certainly possible. Often, these cases of extreme calculus overgrowth appear to be associated with “systemic conditions such as poorly controlled type 2 diabetes” [33]. Although there is no evidence that Pyrrhus suffered from such a systemic condition [2], it remains an intriguing possible explanation for his unusual presentation. Ultimately, given the precedent established in current literature, it appears possible that Plutarch’s description of Pyrrhus’ teeth as one “continuous bone” [5] is a description of a case of dental calculus overgrowth.

**Figure 3:**
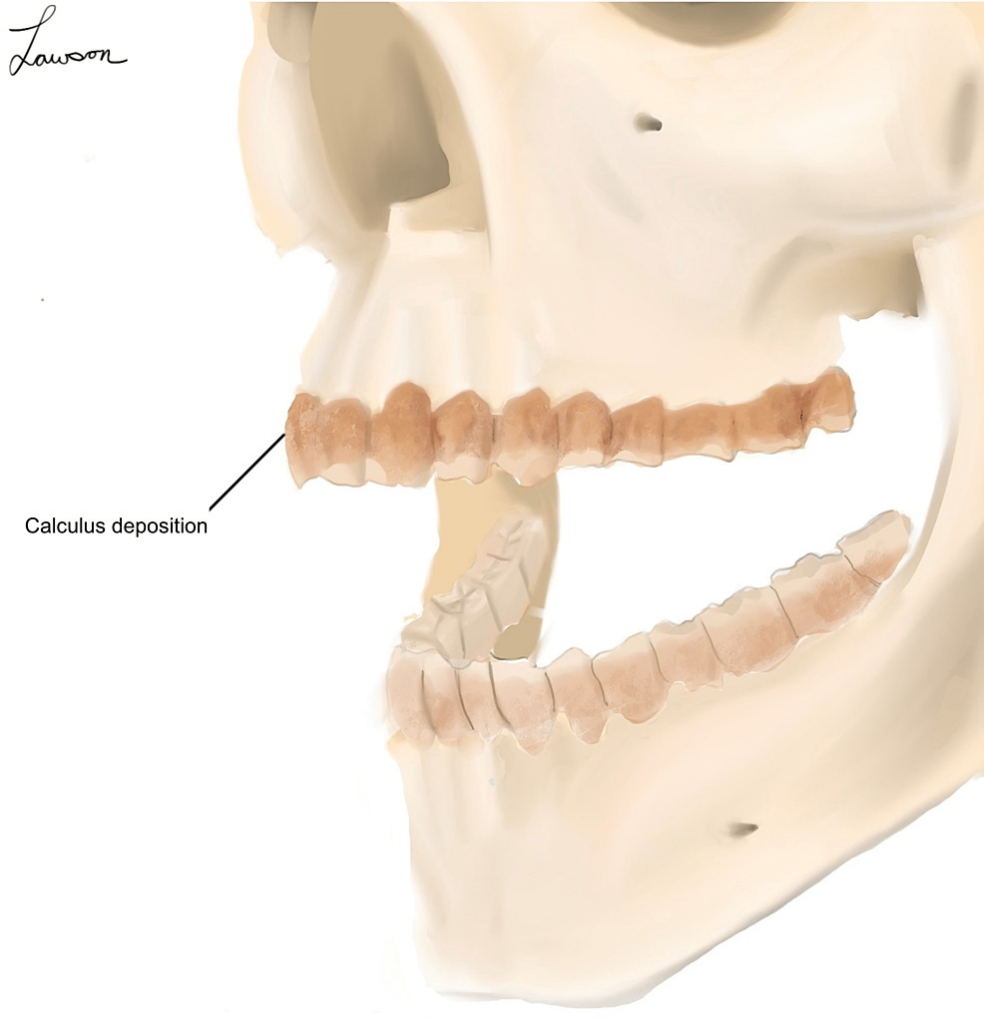
A depiction of dental anatomy with heavy calculus deposition, giving the described appearance of "one continuous bone" along the top row of teeth. This illustration was drawn by Dr. Michael Lawson, the author of this paper.

Given this, a possible presentation of Pyrrhus' teeth would have been consistent with that seen in Figure 3.

## Conclusions

While it is impossible to definitively determine the true cause of Pyrrhus’ dental pathology, we suggest that Plutarch’s description of the king, while possibly based on an exaggeration for propaganda purposes, may have an origin in historical fact. The surviving imagery of Pyrrhus shows evidence of mild frontal bossing, but it is unlikely that the king experienced any systemic syndromes that also caused his unique dental pathology. It is possible that Pyrrhus may have developed his appearance due to an overgrowth of dental calculus. Dental calculus was not unknown to his chronological and cultural contemporaries, and significant overgrowth can be observed in modern examples.
